# Draft Genome Sequence of the Industrially Significant Bacterium Bacillus amyloliquefaciens NRRL 942

**DOI:** 10.1128/MRA.01374-18

**Published:** 2018-11-08

**Authors:** Matthew J. Meier, Annette Dodge, Lee A. Beaudette

**Affiliations:** a Biological Assessment and Standardization Section, Environment and Climate Change Canada, Ottawa, Ontario, Canada; Loyola University Chicago

## Abstract

Bacillus amyloliquefaciens NRRL 942 is a Gram-positive bacterium with several potential industrial uses. We have sequenced the whole genome of this organism to assist in understanding the biological mechanisms that might modulate human health or environmental risk in the event of its release into the environment.

## ANNOUNCEMENT

Bacillus amyloliquefaciens NRRL 942 is a bacterial strain on Canada’s domestic substances list (DSL; a list of substances that were manufactured in, imported into, or used in Canada on a commercial scale prior to 1986 that is available at https://www.canada.ca/en/environment-climate-change/services/canadian-environmental-protection-act-registry/substances-list/domestic.html). This strain (also known as L-A1, originally isolated by G. A. Jeffrey, and known as 13563-0 on the DSL) normally inhabits the rhizosphere in soil, and it is known for its production of the enzyme α-amylase, which is capable of liquefying starch (1, 2). It has applications in bioaugmentation for waste treatment, septic tanks, plant disease management, and a variety of bioremediation and/or composting applications (3). Here, we present the whole-genome sequence of Bacillus amyloliquefaciens NRRL 942.

A glycerol stock of B. amyloliquefaciens strain NRRL 942 was used to inoculate a nutrient agar plate. A single colony was used to inoculate an overnight culture in nutrient broth (37°C with shaking at 220 rpm), and total genomic DNA was isolated using the Wizard genomic DNA purification kit (Promega). Genomic DNA (1 µg) was enzymatically fragmented (10 min) and size selected using the E-Gel Size Select agarose gel to select 300-bp fragments. We constructed a genomic library using the Ion Xpress Plus DNA fragment library kit (Thermo Fisher Scientific) and sequenced it on the Ion Torrent Personal Genome Machine (PGM) platform (Life Technologies).

We obtained 3,105,875 raw reads with an average length of 251 bp (782,212,297 total bases). Reads were processed using the EDGE bioinformatics platform version 2.3.0 (4). Default parameters were used for quality trimming (with the addition of adapter sequence removal of the P1 and A adapter sequences), after which 3,093,720 reads with an average length of 252 bp remained (781,672,016 total bases). Assembly was performed using SPAdes version 3.11.1 with default parameters (5), which produced 77 contigs, with an *N*_50_ of 875,453 bp and a total assembly size of 3,792,988 bp. The mean GC content was 40%. Mapping quality-controlled reads to the contigs using BWA-MEM with default parameters (6) in EDGE revealed an average genome coverage of 205×. Contigs were annotated using the NCBI Prokaryotic Genome Annotation Pipeline (7), resulting in a total of 3,967 genes (3,676 coding).

Bacillus amyloliquefaciens NRRL 942 is closely related to Bacillus amyloliquefaciens LL3, with 98.2% of NRRL 942 reads mapping to the genome of strain LL3 (GenBank accession no. NC_017190), covering 93.6% of the bases with an average coverage of 192× and differing by 376 single-nucleotide polymorphisms (SNPs) and 79 indels. This was determined using read mapping with BWA-MEM (as described above) and SNP/indel calling with the EDGE bioinformatics platform. We identified eight antimicrobial resistance genes in this strain using the Antibiotic Resistance Genes database (http://ardb.cbcb.umd.edu/) through EDGE. This includes five efflux pumps, namely, the ABC transporter system macrolide-lincosamide-streptogramin B efflux (GenPept accession no. NP_388149), MFS antibiotic efflux (GenPept accession no. P96712), tetracycline efflux (GenPept accession no. YP_001966009), and two SMR antibiotic efflux pumps (GenPept accession no. YP_001420884 and YP_001420885). We also identified genes conferring resistance to fosfomycin (GenPept accession no. YP_001420711), penicillins, cephalosporins and carbapenems (β-lactamase Subclass B1 136), and a undecaprenyl pyrophosphate phosphatase (GenPept accession no. YP_001422387). We also found two virulence factors, *capC* (capsule biosynthesis protein, YP_080919) and *tuf* (elongation factor Tu, NP_326236). This genome sequence can be used to guide industrial users of this microorganism in establishing safe practices and to inform regulatory agencies about potential genes of interest that may be used in risk assessment.

### Data availability.

This whole-genome shotgun project has been deposited in GenBank under the accession no. QVEJ00000000 (BioProject accession no. PRJNA486183 and BioSample accession no. SAMN09839534). This announcement describes the first version, QVEJ01000000.
